# Glances at the Hospitals

**Published:** 1900-09-22

**Authors:** 


### GLANCES AT THE HOSPITALS.

### THE LIVERPOOL NORTHERN HOSPITAL.

On January 1st, 1899, this hospital contained 127 patients, and during the year 1,668 were admitted, 1,138 were discharged cured, 329 were relieved, 15 were discharged for other causes, 90 died, 47 were brought in dead, 53 died within 24 hours of admission, and 123 remained under treatment on December 31st. There were 7,411 out-patients, 474 were medical cases, 1,561 surgical, and 5,376 were accidents. The total number of cases treated was 9,206. It is instructive to glance at the nationalities of those treated in the out-patients' department; and it would be more instructive still if the proportion which these various nationalities bear to the total population of the district could be given. The English were represented by 3,823 patients, the Irish by 2,948, the Welsh by 280, the Scotch by 204, and foreign by 156. A very clear analysis of the operations of the year is given, the columns of the table showing the "cured," "relieved," and "died." We notice that the "radical cure" for inguinal hernia was six times performed, and that all were cured. For strangulated hernia there were five operations and one death. There were four cases of appendicitis, and of these three died, but there were seven other cases not operated on. Altogether there were 867 operations, and of these 823 were successful. The medical table is faulty, as it does not state the results obtained. We read that 20 cases of enteric fever were treated, 41 cases of pneumonia, and so on; but we are not told how many recovered, and we lose much of the interest that should be felt when examining the statistics. It would also be useful to have a table showing the kind of anæsthetics used in the 867 operations. The receipts from all sources amounted to £6,469, and the ordinary expenditure to £7,305; and the report states that there is a balance of £3,761 due to the bankers.

### THE BUCKINGHAMSHIRE HOSPITAL AT AYLESBURY.

This report is printed on a folio sheet, and the sooner it can be reduced to pamphlet form the better it will be for everyone who has to touch it. The total income was £2,874; but donations and legacies account for £1,081 of that total, and £629 was derived from interest on investments. The total expenditure was £2,930; but £1,037 of that sum was invested. We did not notice any information regarding the cost per bed occupied, or the weekly cost per head, and the report contains neither medical nor surgical statistics beyond the numbers under treatment. There were 32 patients in the hospital on January 1st, 1899, and 300 were admitted during the year. Of the total, 196 were discharged cured, 67 relieved, 24 for other causes, 14 died, and 31 were under treatment at the end of the year. In the out-patients' department 196 were under treatment at the beginning of 1899, and 1,648 were treated since. Of these, 1,598 were discharged (reasons not given) and 246 remained on the books.

### THE OLDHAM INFIRMARY.

Here the receipts from ordinary sources amounted to £5,224, and from legacies to £1,090. The payments amounted to £6,392, but £1,000 of this was passed on to the special fund for additional accommodation, and £219 to the debit balance. There is an Endowment Fund of £40,623, and it may be said that the finances of the hospital are in a sound condition; but it is clear that an income of £6,000 from ordinary sources will be necessary to keep the hospital up to a high state of efficiency. The total number of patients under treatment during the year was 6,644. The in-patients admitted were 748, and 73 remained from the previous year. Of these 409 were discharged cured, 146 relieved, 20 for other reasons, and 69 died. Of the deaths 30 took place within 24 hours of admission. Three hundred and forty-three operations were performed in the operation theatre, and 300 minor operations were performed in the wards and out-patients' departments. No table showing the results of these operations is given, and the mortality is included in the ordinary tables of the general surgical cases, and although these tables are very well compiled they do not show us all we want to know concerning the major operations.

### THE ROYAL INFIRMARY, HALIFAX.

The income amounted to £13,759, but nearly £7,000 was derived from donations, of which £6,000 was placed to the capital account. The total ordinary expenditure was £7,624, and the balance due to the bank had increased from £714 to £1,540. On January 1st, 1899, the infirmary contained 110 patients, and 1,549 were admitted during the year. Of these 145 were discharged cured, 62 relieved, 26 as incurable, 62 for other reasons, 1,169 were made out-patients, 93 died, and 103 remained under treatment. The duration of each patient's stay in the infirmary was a little over 23 days; the cost of each in-patient was £4 1s. 3d.; the cost per bed occupied was £62 13s. 4d. The total number of out-patients was 6,884, but, in addition, 1,117 casualties were treated, and the average cost of each out-patient was 2s. 4d. The medical and surgical statistics are carefully compiled, and a table of the operations performed during the year is also given. We notice that ovariotomy was performed ten times, and that all were successful. In addition to nearly 300 operations in which details are given, there were 206 minor operations; and also a large number in the eye, ear, and throat department of the infirmary. We venture to suggest in future reports that a short table be introduced showing the kind of anæsthetics used.

